# Molecular and Microscopic Identification of *Sarcocystis* spp. in the Intestines of the Tawny Owl (*Strix aluco*) in Lithuania

**DOI:** 10.3390/ani16132009

**Published:** 2026-07-01

**Authors:** Petras Prakas, Saulius Rumbutis, Viktorija Levinger, Tautvilė Šukytė, Evelina Juozaitytė-Ngugu, Giedrė Pakeltytė, Dalius Butkaukas, Saulius Švažas

**Affiliations:** 1 State Scientific Research Institute Nature Research Centre, Akademijos St. 2, 08412 Vilnius, Lithuania; saulius.rumbutis@gamtc.lt (S.R.); viktorijalevinger20@gmail.com (V.L.); tautvile.sukyte@gamtc.lt (T.Š.); evelina.ngugu@gamtc.lt (E.J.-N.); dalius.butkauskas@gamtc.lt (D.B.); saulius.svazas@gamtc.lt (S.Š.); 2 Tadas Ivanauskas Zoological Museum, Laisvės 106, 44253 Kaunas, Lithuania; giedre.pakeltyte@zoomuziejus.lt

**Keywords:** *Sarcocystis*, *Strix aluco*, *28S* rRNA, *ITS1*, molecular identification, phylogeny, sporocysts, definitive host, prevalence, host–parasite interactions

## Abstract

The role of birds of the Strigiformes order in the transmission of globally distributed *Sarcocystis* parasites remains insufficiently studied. In the present work we aimed to evaluate the diversity of *Sarcocystis* species in the intestines of 22 naturally infected Tawny Owls (*Strix aluco*) sampled in Lithuania. The *Sarcocystis* infection was established using combined light microscopy and DNA sequence analysis. Samples were analyzed using *Sarcocystis*-genus and species-specific primers in nested PCR followed by Sanger sequencing. Sporocysts of *Sarcocystis* spp. were detected in 45.5% of samples, while 77.3% of samples were positive by molecular methods. Comparison of *28S* rRNA and *ITS1* sequences obtained and phylogenetic analyses indicated the presence of eight *Sarcocystis* taxa: *S. halieti*, *S. kutkienae*, *S. turdusi*, and *Sarcocystis* sp. ex *Corvus corax*, associated with birds, and *S. funereus*, *S. glareoli*, *Sarcocystis* sp. LT24Sa1, and *Sarcocystis* sp. LT24Sa11, associated with small mammals. Our results supplement previous findings that some *Sarcocystis* species can be transmitted by birds of three orders: Accipitriformes, Falconiformes, and Strigiformes. Overall, the low burden of sporocysts and predominance of single-species infections (63.6%) suggest that Tawny Owls transmit a limited number of *Sarcocystis* species associated with small mammals and birds.

## Simple Summary

*Sarcocystis* species are apicomplexan parasites infecting reptiles, birds, and mammals. Birds are intermediate hosts for 32 *Sarcocystis* species, and DNA analysis confirms at least 16 species use birds as definitive hosts. Furthermore, birds of the order Stirgiformes serve as definitive hosts for several *Sarcocystis* species. The Tawny Owl (*Strix aluco*), a widespread species in Lithuania and a sedentary predator occupying diverse habitats, represents a potential definitive host for multiple *Sarcocystis* species. However, the diversity of *Sarcocystis* species in the intestines of this Strigiformes bird under natural conditions remains poorly investigated. The study aimed to evaluate the variety of *Sarcocystis* species present in the intestines of naturally infected Tawny Owls using both microscopy and DNA sequencing. Seventeen of the 22 examined Tawny Owl samples tested positive for *Sarcocystis* by molecular analysis, and sporocysts were microscopically observed in 10 of these samples. Molecular identification revealed eight *Sarcocystis* taxa using birds and small mammals as their intermediate hosts. The dominant species detected, *S. glareoli*, forms cysts in the brains of small mammals. The composition of *Sarcocystis* species in the intestines of Tawny Owls corresponds to the dietary patterns reported for this predator in Lithuania.

## 1. Introduction

*Sarcocystis* is one of the most species-diverse and geographically widespread genera within the phylum Apicomplexa [1]. More than 200 *Sarcocystis* species are currently known from reptiles, birds, and mammals. These parasites are distinguished by a two-host life cycle, formation of sarcocysts in muscles of intermediate hosts (IHs) and endogenous sporulation of oocysts in the *lamina propria* of the small intestines of definitive hosts (DHs). Notably, a DH becomes infected through predation or scavenger behavior, by ingesting tissues of the IH containing mature sarcocysts, while an IH acquires infection through the consumption of water or food contaminated with the parasite’s sporocysts. *Sarcocystis* species are described in IHs, in which the pathogenic effects of the parasite may occur [1,2,3]. Additionally, *Sarcocystis* species tend to be less host-specific to their DHs than to their IHs [1].

The main phenotypic criterion of taxonomic value for *Sarcocystis* parasites is the morphological structure of sarcocysts detected in IHs [1,4]. In contrast, oocysts and sporocysts are of no or limited value in discriminating against parasite species in DHs. Notably, there are no structural differences between oocysts and sporocysts of different species [1,4,5,6]. However, slight differences in the morphometric parameters of these parasite stages may occur in DHs [7]. Nevertheless, multiple *Sarcocystis* species infections are commonly established in a single naturally infected DH, and sizes of oocysts and sporocysts of distinct *Sarcocystis* species often overlap, making morphological discrimination of parasite species barely possible [5,6,7,8,9]. Therefore, molecular methods are increasingly being applied to identify *Sarcocystis* species in intestines or fecal samples of predators or scavengers. It is worth mentioning that investigations in which both intestines and feces of the same DH species have been examined demonstrated a higher level of detection of *Sarcocystis* spp. in the intestines [9,10,11,12].

According to the latest literature review, birds serve as IHs of 32 *Sarcocystis* species; furthermore, DNA analysis confirmed at least 16 *Sarcocystis* species in birds as their DHs [1,4,7,13]. In comparison to more widely examined birds of the Accipitriformes order [4,6,7,13,14,15,16,17], the role of birds of the Strigiformes order has still been poorly studied. Based on transmission experiments, Strigiformes birds were confirmed to be DHs of *S. dispersa* [18], *S. espinosai* [19], *S. funereus* [20], *S. rauschorum* [21], *S. sebeki* [22], and *S. strixi* [23]. All these species were shown to be associated with small mammals. Furthermore, *Sarcocystis* sp. sporocysts and oocysts were found in the scrapings of the small intestine of the Tawny Owl (*Strix aluco*), which was fed muscles of the least weasel (*Mustela nivalis*) heavily infected with sarcocysts [24]. Moreover, molecular evidence obtained from naturally infected Strigiformes supports the hypothesis that these raptorial birds may serve as DHs for *S. columbae* and *S. cornixi* transmitted through avian IHs [17], in addition to *S. funereus* [20], *Sarcocystis* cf. *strixi* [17], and *Sarcocystis* sp. [25], whose life cycles involve small mammals as IHs.

The Tawny Owl is one of the most widespread and sedentary species of the Strigiformes order in Lithuania, inhabiting a variety of forested and semi-urban environments [25,26]. The breeding population is estimated at about 4000 pairs [25]. This species is characterized by strongly sedentary behavior, occupying a relatively small core area of up to 50 hectares per individual owl [27]. These territories are most often established in deciduous or mixed forests but also frequently extend into urban and other anthropogenic habitats [25,26]. The Tawny Owl is an opportunistic nocturnal predator that primarily feeds on small mammals, particularly rodents (e.g., voles and mice), but also consumes birds, amphibians, and invertebrates [26,28]. A broad trophic niche and frequent predation on potential IHs make the Tawny Owl a suitable candidate as a DH for numerous *Sarcocystis* species. Despite that, as has been described above, under natural conditions the diversity of *Sarcocystis* species infecting the intestines of birds of the Strigiformes order, including Tawny Owls, remains poorly examined.

In the present study, we aimed to investigate the diversity of *Sarcocystis* species in the intestines of naturally infected Tawny Owls using microscopic examination and DNA sequence analysis, with the objective of elucidating host–parasite interactions, trophic transmission pathways, and evolutionary trends within the genus *Sarcocystis*.

## 2. Materials and Methods

### 2.1. Sample Collection and Processing

A total of 22 Tawny Owls were collected between 2014 and 2024 in different districts of Lithuania. All birds used in this study were collected postmortem due to collisions with motor vehicles, power lines, buildings, and similar causes. Specimens were obtained from the Kaunas Tadas Ivanauskas Zoology Museum, which serves as the Lithuanian national authority for monitoring dead birds. Birds were delivered to the Laboratory of Molecular Ecology, State Scientific Research Institute Nature Research Centre, Vilnius, Lithuania, for detailed morphological and molecular analysis. The samples were transported in biohazard and transport bags under established temperature protocols at −20 °C to maintain sample integrity and safety. The current investigation was approved by the Animal Welfare Committee of the Nature Research Centre (no. GGT-9, issued on 12 January 2024).

### 2.2. Preparation of Intestine Samples and Microscopical Examination of Sarcocystis spp.

Isolation of *Sarcocystis* spp. from the intestines of each Tawny Owl was performed by applying a modified technique based on Verma et al. [29]. This approach allows for the recovery of multiple developmental stages of *Sarcocystis* spp., including sporocysts, oocysts, and sporulating oocysts. Intestinal mucosal scrapings were collected following a longitudinal incision of the intestine. The collected epithelial material was immersed in 50 mL of distilled water (dH_2_O) and homogenized using a commercial mixer at high speed for 1–2 min. The homogenate was then centrifuged at 1600 rpm for 6 min at 20 °C, after which the supernatant was carefully removed. The remaining sediment was resuspended in 50 mL of distilled water. The steps of homogenization, centrifugation, and washing were repeated until the supernatant remained clear. The resulting sediment was then diluted with 20 mL of Hank’s Balanced Salt Solution (HBSS) and 20 mL of 5.25% sodium hypochlorite (bleach) solution and incubated in an ice bath for 30 min. Following incubation, the sample was centrifuged, and the partially decanted supernatant was replaced with dH_2_O. This washing step was repeated until the odor of sodium hypochlorite was no longer detectable. The resulting sediment was examined under a light microscope (LM) at ×400 magnification for the presence of *Sarcocystis* spp. oocysts and sporocysts. Parasite load was estimated by counting the number of oocysts and sporocysts present in 20 µL of sediment spread beneath a 24 × 24 mm coverslip. Morphological measurements were performed, and results were presented as mean ± standard deviation (SD). At the final stage, 400 μL of the resuspended sediment from each sample was used for DNA extraction. All samples underwent this step regardless of whether *Sarcocystis* spp. oocysts and sporocysts were detected.

### 2.3. Molecular Analysis of Sarcocystis spp.

Genomic DNA extraction of *Sarcocystis* spp. from intestinal scapings of Tawny Owls was performed using a GeneJET Genomic DNA Purification Kit (Thermo Fisher Scientific Baltics, Vilnius, Lithuania). Purified genomic DNA was stored at −20 °C for further molecular manipulation. Detection of *Sarcocystis* spp. was performed using nested PCR (nPCR) to enable amplification of low quantities of parasite DNA. Subsequently, all amplified products were subjected to Sanger sequencing for the species identification.

The identification of *Sarcocystis* spp. employing birds as IHs was carried out using the amplification of *ITS1*. At the first round of nPCR SU1F forward 5′-GATTGAGTGTTCCGGTGAATTATT-3′ and 5.8SR2 reverse 5′-AAGGTGCCATTTGCGTTCAGAA-3′ were applied [30], while species-specific primers were used in the second round of nPCR. Overall, 10 *Sarcocystis* species with birds as IHs (*S. calchasi*, *S. columbae*, *S. cornixi*, *S. corvusi*, *S. fulicae*, *S. halieti*, *S. kutkienae*, *S. lari*, *S. turdusi* and *S. wobeseri*) were tested (Table 1). The *ITS1* region was employed for the screening of *Sarcocystis* spp. in birds because previous studies have shown that, among the molecular markers currently available, it offers the greatest resolution for distinguishing relatively recently evolved avian *Sarcocystis* species [4,31].

The differentiation of *Sarcocystis* spp. with small mammals as IHs was carried out using *Sarcocystis* genus- and species-specific approaches, thereby increasing the probability of detecting species. Two *Sarcocystis* species (*S. funereus* and *S. strixi*) were screened using an external Sgrau281/Sgrau282 (Sgrau281 forward 5′-GAACAGGGAAGAGCTCAAAGTG-3′ and Sgrau282 reverse 5′-GGTTTCCCCTGACTTCATTCTAC-3′) [32] primer pair. In the meantime, species-specific primers targeting *28S* rRNA were used in the second round of nPCR (Table 1). Likewise, *S. glareoli* was tested using genus-specific and species-specific primers in the first and second round of nPCR, respectively. However, in the case of *S. glareoli* identification, two sets of primers amplifying *28S* rRNA and *ITS1* were used. Furthermore, two genus-specific primer pairs SgraupaukF/SgraupaukR and Ssprod2R/Ssprod2F serving as internal primers were used in the combination with Sgrau281 and Sgrau282 external primers. The choice of genetic regions for the screening of small mammals’ *Sarcocystis* spp. is outlined below. Previous studies have demonstrated that the *28S* rRNA and *ITS1* regions are the most suitable genetic markers for the identification of *Sarcocystis* spp. employing small mammals as IHs [33,34]. However, to date, only a limited number of *ITS1* sequences are available for *Sarcocystis* species with a bird–small mammal (DH–IH) life cycle [6,16]. Nuclease-free water instead of template DNA was used as the negative control in both rounds of nPCR steps. A dataset of *Sarcocystis* species DNA, isolated from individual sarcocysts and identified based on sequencing data, including all tested species except *S. strixi*, was taken from the collection of the Molecular Ecology Laboratory of the State Scientific Research Institute Nature Research Centre.

PCRs were conducted under the conditions described previously [6] and using annealing temperatures provided in Table 1.

The visualization, purification, and sequencing of amplified products were carried out using a previously described protocol [9]. Only pure sequences that did not contain double peaks or polysignals were included in further analysis. The obtained sequences were compared with various *Sarcocystis* spp. that are available in NCBI GenBank using the nucleotide BLAST (http://blast.ncbi.nlm.nih.gov/, accessed on 5 May 2026). The *Sarcocystis* spp. sequences generated in the current work were submitted to GenBank with the accession numbers PZ429785–PZ429814 and PZ429815–PZ429830.

### 2.4. Phylogenetic and Diversity Analysis

Phylogenetic analyses were carried out using MEGA v.12.0.14 [35]. Multiple alignments of *28S* rRNA and *ITS1* sequences were created using the ClustalW algorithm. Phylogenetic trees were generated using the maximum likelihood method. The nucleotide substitution model best fitting to each of dataset analyzed was chosen based on the lowest values of the Bayesian Information Criterion (BIC). Three taxa, *Eumonospora henryae*, *Cystoisospora yuensis* and *Isospora rastegaievae*, showing the highest sequence similarity to our sequences excluding *Sarcocystis* spp., were set as the outgroup for *28S* rRNA analyses. Meanwhile, *Sarcocystis lari* was used as the outgroup for *ITS1* phylogenetic trees. The robustness of the phylogenetic tree was assessed by bootstrap analysis with 1000 replicates.

For the comparison of *Sarcocystis* spp. diversity in Tawny Owl samples with that previously reported in Common Buzzard [16], Eurasian Sparrowhawk, and Northern Goshawk [6] samples from Lithuania, species richness (S) and Shannon’s diversity index (H) were calculated using PAST v. 5.0.2 [36].

## 3. Results

### 3.1. Detection of Sarcocystis spp. Oocysts and Sporocysts by Light Microscopy

**Figure 1:**
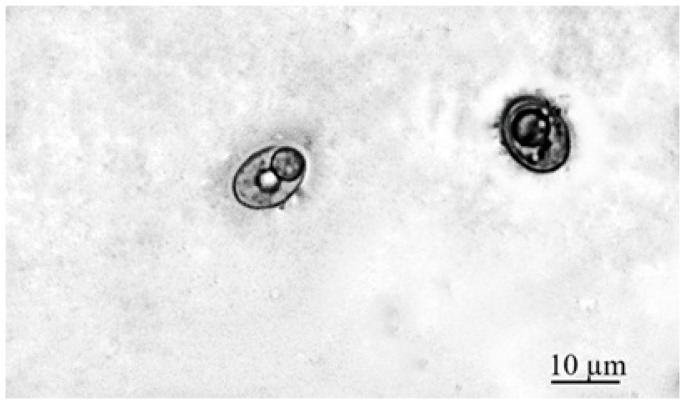
Microscopic characterization of sporocysts of *Sarcocystis* spp. detected in intestinal scrapings of Tawny Owls collected in Lithuania.

Light microscopic examination of intestinal epithelial scrapings revealed infection in 10/22 (45.5%) Tawny Owls. Sporocysts were observed sporadically, with the number detected within a 24 × 24 mm coverslip area ranging from one to 10 per sample. The sporocysts were ellipsoidal in shape (Figure 1). Their sizes were in the range of 11.68–14.55 × 8.74–10.07 µm (12.67 ± 1.12 × 9.99 ± 0.86 µm; *n* = 24). No oocysts were observed in any of the examined birds.

### 3.2. Genetic Identification of Sarcocystis Species

Overall, 46 pure high-quality DNA sequences were generated in this study, including 30 *28S* rRNA and 16 *ITS1* sequences. Excluding primer-binding sites, the length of sequences obtained ranged from 400 bp to 619 bp. Based on the BLAST analysis, the highest similarity matches corresponded to eight different *Sarcocystis* species. Four of these, *S. halieti*, *S. kutkienae*, *S. turdusi*, and *Sarcocystis* sp. ex *Corvus corax*, are known to employ birds as their IHs [4]. Two species, *S. glareoli* [37] and *S. funereus* [20], were previously reported to be associated with small mammals. Furthermore, two genetically new lineages, *Sarcocystis* sp. LT24Sa1 and *Sarcocystis* sp. LT24Sa11, were established (Table 2). Both genetic lineages were detected using three primer pairs (SgraupaukF/SgraupaukR, Ssprod2F/Ssprod2R, and SfunerF/SfunerR). Depending on the primers used, the sequences of these lineages showed the highest similarity (97.67–98.38% and 97.73–98.44%, respectively) to *S. glareoli*, *S. jamaicensis*, *Sarcocystis* sp. Rod3, and *Sarcocystis* sp. RodES1Av. It should be noted that the intraspecific and interspecific similarity values did not overlap for any of the detected *Sarcocystis* species, supporting the reliability of the species identification. However, only minor differences were observed between the *28S* rRNA sequences of *S. glareoli* and *S. halieti* obtained in this study and those of their closely related species.

Three *Sarcocystis* species with birds as IHs, *S. halieti*, *S. kutkienae*, and *S. turdusi*, were identified using species-specific primers targeting *ITS1*. Meanwhile, the fourth species using birds as IHs, *Sarcocystis* sp. ex *Corvus corax*, was confirmed using the SgraupaukF/SgraupaukR primer pair targeting the *28S* rRNA of multiple *Sarcocystis* spp. Other bird-associated *Sarcocystis* species tested, i.e., *S. calchasi*, *S. columbae*, *S. cornixi*, *S. corvusi*, *S. fulicae*, *S. lari*, and *S. wobeseri*, were not detected using species-specific primers.

Two pairs of primers, GsSglaF1/GsSglaR1 and GsSglajamF1/GsSglajamR1, were found to be specific for the amplification of *S. glareoli*. In contrast, SfunerF/SfunerR, designed to identify *S. funereus*, also amplified *Sarcocystis* sp. LT24Sa1 and *Sarcocystis* sp. LT24Sa11. Moreover, GsSstrF1/GsSstrR1, developed in silico for the amplification of *S. strixi*, amplified the DNA of *S. glareoli* and *S. halieti*. Of the two primer pairs (SgraupaukF/SgraupaukR and Ssprod2F/Ssprod2R) designed to amplify multiple *Sarcocystis* spp., SgraupaukF/SgraupaukR targeted a broader range of species and samples analyzed.

Overall, the molecularly based detection rate of *Sarcocystis* spp. in samples examined was 77.3%, with 17 samples testing positive by nested PCR following Sanger sequencing (Table 3). Five birds were negative by both morphological and molecular analysis. Notably, seven samples were positive only by molecular investigations, whereas no samples were positive exclusively by light microscopy. Among the eight *Sarcocystis* taxa identified, three (*S. funereus*, *Sarcocystis* sp. LT24Sa1, and *Sarcocystis* sp. LT24Sa11) were found only using DNA analysis. In contrast, *S. glareoli*, *S. halieti*, *S. kutkienae*, *S. turdusi*, and *Sarcocystis* sp. ex *Corvus corax* were detected in samples of Tawny Owls, which had *Sarcocystis* sporocysts/oocysts confirmed by LM.

### 3.3. Phylogenetic Relationship of Sarcocystis Species Identified

**Figure 2:**
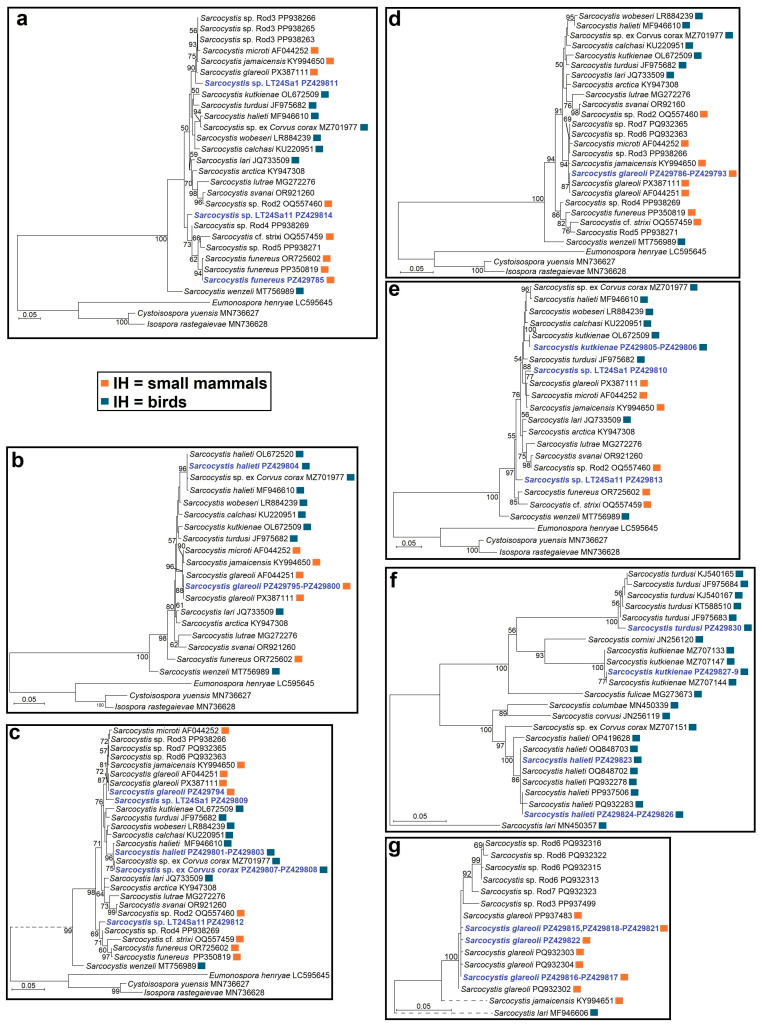
Maximum likelihood phylogenetic trees of selected *Sarcocystis* spp. based on *28S* rRNA (**a**–**e**) and *ITS1* sequences (**f**,**g**). Sequences were obtained using SfunerF/SfunerR (**a**), GsSstrF1/GsSstrR1 (**b**), SgraupaukF/SgraupaukR (**c**), GsSglaF1/GsSglaR1 (**d**), Ssprod2F/Ssprod2R (**e**), GsSturF/GsSturR, GsShalF/GsShalR2 and GsSkutkF2/GsSkutkR2 (**f**) and GsSglajamF1/GsSglajamR1 (**g**) primer pairs. Kimura 2-parameter + G + I (**a**), HKY + I (**b**), HKY + G + I (**c**–**e**), and HKY + G (**f**) and Tamura 3-parameter (**g**) evolutionary models were set for analyses. Bootstrap values > 50 are presented next to branches. Sequences generated in the present study are displayed in indigo. Only different genotypes of the same species were used in analyses. The dashed line indicates that its length does not correspond to the evolutionary distance.

Phylogenetic trees were generated based on the primers used for the amplification of sequences obtained (Figure 2). Regarding the *28S* rRNA, two species, *S. funereus* and *S. glareoli*, identified with species-specific primers, were placed with conspecific sequences with high support (94 and 87 bootstrap values) (Figure 2a,d). The first of the two genetically new lineages, *Sarcocystis* sp. LT24Sa1, was placed together with *S. glareoli*, *S. jamaicensis*, *S. microti*, *Sarcocystis* sp. Rod3, *Sarcocystis* sp. Rod6 and *Sarcocystis* sp. Rod7, forming a separate well-supported branch in this cluster of closely related taxa (Figure 2a,c,e). In two phylogenetic trees, the phylogenetic position of the second genetically new lineage, *Sarcocystis* sp. LT24Sa11, was not significantly resolved (Figure 2a,e), while in the third phylogenetic tree, this lineage was most closely related to *S. funereus*, *Sarcocystis* cf. *strixi*, and *Sarcocystis* sp. Rod4 (Figure 2c). The *28S* rRNA sequences of *S. kutkienae* (Figure 2e) and *Sarcocystis* sp. ex *Corvus corax* (Figure 2c) significantly grouped with other isolates of certain species, while the analyzed sequences of *S. halieti* could not be discriminated from those of *Sarcocystis* sp. ex *Corvus corax* (Figure 2b).

Based on *ITS1*, *S. halieti*, *S. kutkienae*, and *S. turdusi* sequences generated using species-specific primers, with maximum support, were grouped with other sequences of the same species (Figure 2f). Finally, our *ITS1* sequences of *S. glareoli* were placed in one cluster together with those of *S. glareoli* retrieved from GenBank and those of *Sarcocystis* sp. Rod3, *Sarcocystis* sp. Rod6, and *Sarcocystis* sp. Rod7 (Figure 2g). Within this cluster *Sarcocystis* sp. Rod6 and *Sarcocystis* sp. Rod7 were highly supported sister taxa, while phylogenetic analysis could not reliably differentiate sequences of *S. glareoli* from that of *Sarcocystis* sp. Rod3.

### 3.4. Distribution of Detected Sarcocystis spp.

**Figure 3:**
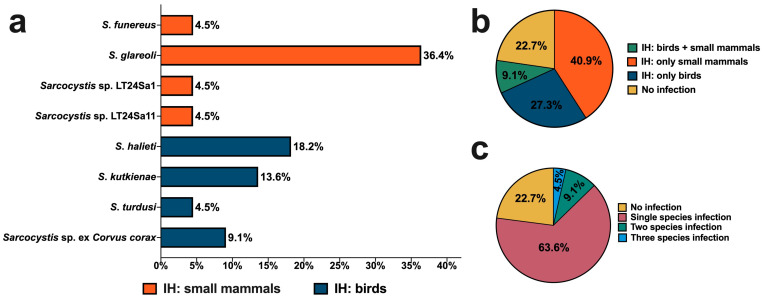
The distribution patterns of the molecularly detected *Sarcocystis* species in intestinal scrapings of analyzed Tawny Owls from Lithuania. (**a**) Detection percentage of certain *Sarcocystis* species based on *28S* rRNA and *ITS1* sequence data. (**b**) The distribution of *Sarcocystis* spp. with small mammals and birds as their IHs. (**c**) Number of *Sarcocystis* species determined per sample.

The distribution pattern of eight molecularly identified *Sarcocystis* species is depicted in Figure 3. *Sarcocystis glareoli* was the most frequently detected species, occurring in 36.4% of samples. The DNA of *S. halieti*, *S. kutkienae*, and *Sarcocystis* sp. ex *Corvus corax* with birds as IHs was confirmed in four, three, and two birds, respectively. Finally, four species, *S. funereus*, *S. turdusi*, *Sarcocystis* sp. LT24Sa1, and *Sarcocystis* sp. LT24Sa11, were detected in a single sample each (Figure 3a). The highest proportion of samples, 40.9%, contained *Sarcocystis* spp. utilizing only small mammals as their IHs, followed by 27.3% of samples encompassing *Sarcocystis* spp. with exclusively birds as their IHs, while in two samples (9.1%), parasite species forming sarcocysts in tissues of small mammals and birds were found (Figure 3b). Single-species infection was most commonly (63.6%) observed, while co-infections were significantly rarer, comprising 13.6% (Figure 3c). Co-infections involving two *Sarcocystis* species were observed in two birds, whereas a co-infection with three species was detected in one sample. Both two-species infections were composed of one species that uses small mammals as its IH and another that employs birds as their IHs. Three-species infections were comprised of *Sarcocystis* spp. with birds as their IHs (*S. halieti*, *S. turdusi*, and *Sarcocystis* sp. ex *Corvus corax*) (Table 3).

## 4. Discussion

### 4.1. Diversity of Sarcocystis spp. Using Tawny Owl as Definitive Host

Until now, limited data have been accumulated on the role of the Tawny Owl in the transmission of *Sarcocystis* species [1]. Laboratory experiments have shown that Tawny Owl is a DH of *S. sebeki* and *S. scotti*, which both use the house mouse (*Mus musculus*) as their IH [1,22,38,39]. However, currently *S. sebeki* is considered to be an invalid species [1]. In addition, *Sarcocystis* sp. employing the least weasel as its IH was found in the intestines of a Tawny Owl [24]. The current work presents the first molecular identification of eight *Sarcocystis* taxa (*S. funereus*, *S. glareoli*, *S. halieti*, *S. kutkienae*, *S. turdusi* and *Sarcocystis* sp. ex *Corvus corax*, *Sarcocystis* sp. LT24Sa1 and *Sarcocystis* sp. LT24Sa11) in intestinal scrapings of naturally infected Tawny Owls. Of these *Sarcocystis* species, only *S. funereus* was previously recorded in members of the family Strigidae [20], while none of them were confirmed in the Tawny Owl. So, the present study provides the first molecularly supported host record of eight *Sarcocystis* taxa in Tawny Owls. Notably, three taxa (*S. funereus*, *Sarcocystis* sp. LT24Sa1, and *Sarcocystis* sp. LT24Sa11) were detected exclusively by molecular methods, whereas five taxa (*S. glareoli*, *S. halieti*, *S. kutkienae*, *S. turdusi*, and *Sarcocystis* sp. ex *Corvus corax*) were associated with samples in which *Sarcocystis* oocysts/sporocysts had been confirmed by light microscopy. On the one hand, the use of DNA analysis for all samples, irrespective of the detection of oocysts/sporocysts of *Sarcocystis*, is a desirable approach, as molecular techniques enable the detection of taxa that may be overlooked by light microscopy due to low parasite burden. On the other hand, the detection of *Sarcocystis* spp. DNA alone does not necessarily indicate that the animals studied are DHs for the identified *Sarcocystis* species. The detected genetic material may originate from parasites present in ingested prey rather than from active infection in the DH [5,40]. Nevertheless, it cannot be excluded that some *Sarcocystis* species found in the intestinal scrapings of raptors represent cases of pseudoparasitism rather than true infection [41,42]. For example, *S. kutkienae* and *Sarcocystis* sp. ex *Corvus corax*, both of which utilize corvids as IHs, may be capable of completing either diheteroxenous or dihomoxenous life cycles [1,43]. Corvids are known to be IHs of three *Sarcocystis* spp. identified in the present study (*S. halieti*, *S. kutkienae*, and *Sarcocystis* sp. ex *Corvus corax*) [43]. *Sarcocystis kutkienae* and *Sarcocystis* sp. ex *Corvus corax* form sarcocysts in corvid muscles, whereas *S. halieti* utilizes a wide range of avian IHs across several orders [4,43].

Furthermore, several *Sarcocystis* species, including *S. alces*, *S. capreolicanis*, *S. gracilis*, *S. tenella*, and *S. halieti*, were recently identified in the intestinal scrapings of brown rats (*Rattus norvegicus*) [44]. It is known that the first four species are transmitted by carnivorous mammals [1]. Consequently, the involvement of brown rats as DHs for these *Sarcocystis* spp. appears improbable. Nevertheless, when considering the role of Tawny Owls in the transmission of certain *Sarcocystis* species, it is important to take into account trophic transmission pathways. All eight detected *Sarcocystis* taxa are associated with either small mammals or birds, so the assumption that these *Sarcocystis* spp. use Tawny Owls as their true DHs is biologically plausible. However, transmission experiments are needed for the conclusive confirmation of whether identified *Sarcocystis* species can employ Tawny Owls as DHs.

### 4.2. Host Specificity of Sarcocystis spp. Transmittable via Birds

*Sarcocystis* species typically exhibit a higher degree of specificity toward their IHs, whereas their DHs range tends to be broader [1,45]. A classic example illustrating this statement is the species *S. cruzi*, which parasitizes cattle and bison [46,47] and can be transmitted by members of the Canidae and Mustelidae families [12,48,49]. By contrast, some species, like *S. neurona* and *S. canis*, are considered to have multiple IHs from taxonomically diverse species [1,50,51,52,53]. Thus, *Sarcocystis* species differ in a range of biological characteristics, including host specificity, and any generalization should be made with cautiously, taking into account the host groups under consideration [1].

Six *Sarcocystis* species, *S. calchasi*, *S. columbae*, *S. falcatula*, *S. halieti*, *S. kirmsei*, and *S. wobeseri*, form sarcocysts in muscles of birds belonging to at least two different orders [4]. Furthermore, to date, nine *Sarcocystis* species (*S. calchasi*, *S. columbae*, *S. cornixi*, *S. corvusi*, *S. halieti*, *S. kutkienae*, *S. lari*, *S. turdusi*, and *S. wobeseri*) are known that have been genetically identified in naturally infected birds as both IHs and DHs [1,4,43]. Among these species, *S. columbae* and *S. halieti* have been detected in more than 10 DH species. In addition, *S. columbae* and *S. cornixi* have been confirmed in DHs belonging to three different families: Accipitridae, Falconidae, and Strigidae [4,17]. Additionally, in the current study we identified *S. halieti* in Tawny Owls, which was previously found in Accipitriformes and Falconiformes [4,6,16,17]. Furthermore, the most prevalent *Sarcocystis* species in the intestines of Tawny Owls was *S. glareoli* (36.4%), previously detected in intestinal samples of the Common Buzzard (*Buteo buteo*) [15], Red-shouldered Hawk (*Buteo lineatus*) [14], Northern Goshawk (*Accipiter gentilis*) [17], Eurasian Sparrowhawk (*Accipiter nisus*) [17], Griffon Vulture (*Gyps fulvus*) [17] (Accipitridae) and Common Kestrel (*Falco tinnunculus*) [17] (Falconidae). Notably, before 2022 it was generally supposed that the sole DH of *S. glareoli*, forming brain cysts in the small mammals, mainly voles, is the Common Buzard [1,15]. So, our results confirm that some *Sarcocystis* species might be transmittable via members of orders Accipitriformes, Falconiformes and Strigiformes. Moreover, the study of predator intestinal contents and feces helps to clarify *Sarcocystis* diversity and host specificity.

### 4.3. Identification of Two New Sarcocystis Genetic Lineages in Tawny Owls

In the present study two genetically new *Sarcocystis* lineages, *Sarcocystis* sp. LT24Sa1 and *Sarcocystis* sp. LT24Sa11, were detected. Each of these two lineages were detected in samples in which oocysts/sporocysts of *Sarcocystis* were not observed. However, the DNA of both lineages was amplified with the help of three different pairs of primers (SgraupaukF/SgraupaukR, Ssprod2F/Ssprod2R, and SfunerF/SfunerR), strengthening the evidence of the nonaccidental presence of these lineages in samples examined. Phylogenetic analysis demonstrated that *Sarcocystis* sp. LT24Sa1 forms a well-supported sister branch to a cluster consisting of the small-mammal brain-cyst-producing *S. glareoli* and *S. microti* [1,54,55] and to them closely related *S. jamaicensis*, *Sarcocystis* sp. Rod3, *Sarcocystis* sp. Rod6, and *Sarcocystis* sp. Rod7 (Figure 2). Three obtained *28S* rRNA sequences of *Sarcocystis* sp. LT24Sa1 sharing ≤ 98.38% similarity compared to other *Sarcocystis* spp. (Table 2). It is very likely that *Sarcocystis* sp. LT24Sa1 presents a distinct *Sarcocystis* species, as previous findings showed minor variation of up to 1% between *S. glareoli*, *S. jamaicensis*, *S. microti*, *Sarcocystis* sp. Rod3, *Sarcocystis* sp. Rod6 and *Sarcocystis* sp. Rod7 [37]. However, our 616–619 bp *28S* rRNA sequences of *Sarcocystis* sp. LT24Sa1 were 100% identical compared with the 304–316 bp sequences of *Sarcocystis* sp. from the intestines of Red-shouldered Hawks (OK576425, OK576459, OK576461, and OK576462) and Red-tailed Hawks (*Buteo jamaicensis*) (OK576433) in the USA [14]. Thus, it is highly likely that species belonging to the *Sarcocystis* sp. LT24Sa1 genetic lineage occur in *Buteo* hawks.

Based on *28S* rRNA, another *Sarcocystis* genetic lineage identified in the study, *Sarcocystis* sp. LT24Sa11, exhibited the highest similarity with *S. glareoli*, 97.52–98.44%. Nevertheless, phylogenetic analyses indicated close relationships of *Sarcocystis* sp. LT24Sa11 with *S. funereus*, *Sarcocystis* cf. *strixi* and *Sarcocystis* sp. Rod4 (Figure 2c). *Sarcocystis funereus* is cycling between Tengmalm’s Owl (*Aegolius funereus*) (Strigidae family) and small mammals [20]. DNA analysis revealed that *Sarcocystis* cf. *strixi* is using small mammals and birds of prey of the families Accipitridae, Falconidae and Strigidae as their IHs and DHs, respectively [17,23]. Finally, *Sarcocystis* sp. Rod4 was molecularly identified in the intestines of the Common Buzzard and Rough-legged Buzzard (*Buteo lagopus*) [15]. The repeated identification of new *Sarcocystis* genetic lineages associated with small mammals through the study of raptors demonstrates a high diversity and insufficient study of the *Sarcocystis* species that the infect extra-intestinal tissue of small mammals [6,14,15,16,17,20].

### 4.4. The Role of the Tawny Owl in Distributing Sarcocystis spp.

In the present study, based on DNA analysis, eight *Sarcocystis* taxa were identified, four each associated with birds and small mammals. However, a higher proportion of Tawny Owls harbored *Sarcocystis* spp. using only small mammals (50.0%) instead of birds (31.8%) as their IHs. Among the analyzed intestinal samples, single *Sarcocystis* species infections predominated, accounting for 63.6% of cases (Figure 3). Previously in Lithuania, intestinal scrapings of the Northern Goshawk, Eurasian Sparrowhawk, and Common Buzzard were examined for *Sarcocystis* spp. [6,15,16]. The results obtained cannot be directly compared due to the slightly different sets of primers used. However, the same ten species-specific primer pairs targeting *ITS1* of avian *Sarcocystis* spp. were applied. Based on findings using these primers, the lowest prevalence (31.8%) was estimated for Tawny Owl samples, compared to 50.0%, 68.8%, and 81.3% accounted for the Common Buzzard [16], Eurasian Sparrowhawk, and Northern Goshawk [6]. Species richness (S) and diversity (Shannon’s H) followed a similar pattern, with the Northern Goshawk showing the highest values (S = 8; H = 1.90), followed by the Eurasian Sparrowhawk (S = 4; H = 1.20) and the Common Buzzard (S = 5; H = 1.21), while the lowest diversity was observed in the Tawny Owl (S = 3; H = 0.97). These findings correspond to the results of dietary studies of Tawny Owls. In Lithuania small mammals (mostly *Microtus* voles) form more than 60% of prey items identified in Tawny Owl diet, while birds (mostly small passerines but also corvids) form about 30% [26]. Passerine birds play an important role as alternative prey for owls during periods of low vole abundance or environmental stress [56]. The low prevalence of *Sarcocystis* spp. in Tawny Owls can possibly be associated with the strictly sedentary pattern of this species compared to the migratory or partially migratory patterns of the Common Buzzard, Eurasian Sparrowhawk, and Northern Goshawk [57,58].

In this study we identified *S. glareoli* in 36.4% of birds examined, and this *Sarcocystis* species was dominant among the detected *Sarcocystis* spp., which are associated with small mammals. For a long time, it was considered that the bank vole (*Clethrionomys glareolus*) and Common Buzzard are the sole IH and DH of this *Sarcocystis* species [1]. However, cysts of *S. glareoli* were found in the brains of several species of small mammals [37,54,59]. Extensive studies conducted in Lithuania have shown that the main IH of *S. glareoli* is in fact the bank vole [15,37,54,55]. In addition, the parasite was confirmed by means of DNA sequence analysis in the yellow-necked mouse (*Apodemus flavicollis*) and the common shrew (*Sorex araneus*) [37,60]. Furthermore, DNA-based data recently indicated that *S. glareoli* can be transmittable via several different raptor species [17]. Notably, the bank vole represents an important prey species of the Tawny Owl in Lithuania, accounting for 24.6% of all prey items identified in its diet analyses in certain regions [28].

Thus, the relatively high detection rate of *S. glareoli* in samples analyzed is in agreement with the diet peculiarities of the Tawny Owl. In general, the low burden of sporocysts of *Sarcocystis* spp. in intestinal samples of Tawny Owls, together with the predominance of single-species infections, suggests that this raptor is involved in the transmission of a limited number of *Sarcocystis* species associated with small mammals and birds.

## 5. Conclusions

This study provides the first molecular evidence of *Sarcocystis* diversity in the intestines of naturally infected Tawny Owls. Eight *Sarcocystis* taxa associated with birds (*S. halieti*, *S. kutkienae*, *S. turdusi*, and *Sarcocystis* sp. ex *Corvus corax*) and small mammals (*S. funereus*, *S. glareoli*, *Sarcocystis* sp. LT24Sa1, and *Sarcocystis* sp. LT24Sa11) were identified, and all detected species represent the first records in this host. Notably, *S. glareoli* was the most prevalent species, which is known to form cysts in the brains of voles and other small mammals, indicating a broader host range among raptors than previously reported.

Overall, the distribution of detected *Sarcocystis* species corresponds to the diet of Tawny Owls in Lithuania, which is dominated by small mammals and supplemented by birds. The low parasite burden and predominance of single-species infections suggest that Tawny Owls contribute to the transmission of a limited subset of *Sarcocystis* species. The present findings emphasize the importance of integrating molecular and ecological data to better understand host specificity, transmission dynamics, and *Sarcocystis* diversity in birds of prey.

## Figures and Tables

**Table 1:** *Sarcocystis* genus- and species-specific oligonucleotides used in the second round of nPCR for the amplification of target and other *Sarcocystis* spp.

Locus	Primer Pair	F/R	Primer Sequence (5′-3′)	Target Species	T_m_	Ref.
**Sgrau281/Sgrau282 primer pair was used for the first step of nPCR**						
*28S* rRNA	SfunerF SfunerR	F R	CGATTGGAACCTTTTAGAATCC GGTGCAGAGTATAACATCCCTTT	*S. funereus*	57 °C	PS
*28S* rRNA	GsSglaF1 GsSglaR1	F R	GCAAAATGTGTGGTAAGTTTCACAT CCCTCTAAAAAGATGTTACCCTTCT	*S. glareoli*	61 °C	[15]
*28S* rRNA	GsSstrF1 GsSstrR1	F R	GAAATCGAAGACTCTTGACTGAATC AAGAGAAAGAGTGTAGCCCGATCAT	*S. strixi*	59 °C	PS
*28S* rRNA	SgraupaukF SgraupaukR	F R	CGTATTTGCCCTGTGTCCTT GTCGTAGGTGCAAAGCATAACATC	*Sarcocystis* spp.	55 °C	[16]
*28S* rRNA	Ssprod2F Ssprod2R	F R	GGTGAGAGTCCCGTATTTGCTC GTGCAAAGCATAACATCCTTTTA	*Sarcocystis* spp.	55 °C	PS
**SU1F/5.8SR2 primer pair was used for the first step of nPCR**						
*ITS1*	GsScalF2 GsScalR2	F R	CCTTTTGTAAGGTTGGGGACATA GCCTCCCTCCCTCTTTTTG	*S. calchasi*	55 °C	[6]
*ITS1*	GsScolF GsScolR	F R	ATATGTTCATCCTTTCGTAGCGTTG GCCATCCCTTTTTCTAAGAGAAGTC	*S. columbae*	65 °C	[5]
*ITS1*	GsScornF2 GsScornR2	F R	AGTTGTTGACGTTCGTGAGGTC ACACACTACTCATTATCTCCTACTCCT	*S. cornixi*	61 °C	[5]
*ITS1*	GsScovF GsScovR	F R	TATTCATTCTTTCGGTAGTGTTGAG TTACTCTTTTAACAGCTTCGCTGAG	*S. corvusi*	61 °C	[5]
*ITS1*	GsSfulF GsSfulR	F R	CAAAGATGAAGAAGGTATATACGTGAA CTTTACTCTTGAAGAACGACGTTGA	*S. fulicae*	65 °C	[5]
*ITS1*	GsShalF GsShalR2	F R	GATAATTGACTTTACGCGCCATTAC CCATCCCTTTTTCTAAAGGAGGTC	*S. halieti*	65 °C	[5]
*ITS1*	GsSkutkF2 GsSkutkR2	F R	ACACACGGTCGAGTTGATATGAC TCTTTACCCTTAAACAATTTCGTTG	*S. kutkienae*	61 °C	[5]
*ITS1*	GsSlarF GsSlarR	F R	TTCGTGAGGTTATTATCATTGTGCT GGCGATAGAAATCAAAGCAGTAGTA	*S. lari*	65 °C	[5]
*ITS1*	GsSturF GsSturR	F R	GATTTTTGATGTCCGTTGAAGTTAT CATTCAAATATGCTCTCTTCCTTCT	*S. turdusi*	61 °C	[5]
*ITS1*	GsSwobF GsSwobR2	F R	ATGAACTGCTTTTTCTTCCATCTTT CTCCTCTTGAAGGTGGTCGTGT	*S. wobeseri*	61 °C	[5]
*ITS1*	GsSglajamF1 GsSglajamR1	F R	ATGAACTGCTTTTTCTTCCATCTTT CTCCTCTTGAAGGTGGTCGTGT	*S. glareoli* *	58 °C	[16]

F/R—orientation of primers, forward (F) or reverse (R). T_m_—melting temperature. Ref.—reference. *—also amplifies *Sarcocystis* sp. Rod3, *Sarcocystis* sp. Rod6, *Sarcocystis* sp. Rod7 [16,17]. PS—present study.

**Table 2:** Genetic identification of *Sarcocystis* spp. in the analyzed intestinal scrapings of Tawny Owls from Lithuania. Identical or highly similar sequences obtained using the same primer pair were grouped into single entries, with corresponding GenBank accession numbers indicated.

Sarcocystis Species	Region	GenBank Acc No.	Primers	Bp *	Genetic Similarity	
					With Certain Species	With Most Closely Related Species
*S. funereus*	*28S* rRNR	PZ429785	SfunerF/ SfunerR ^SSP^	400	99.50–100%	95.14–95.37% (*S.* sp. Rod4)
*S. glareoli*	*28S* rRNA	PZ429786–PZ429793	GsSglaF1/ GsSglaR1 ^SSP^	515	99.81–100%	99.61% (*S. jamaicensis*, *S.* sp. Rod3, *S.* sp. Rod6, *S.* sp. Rod7, *S.* sp. RodES1Av)
*S. glareoli*	*ITS1*	PZ429815–PZ429822	GsSglajamF1/ GsSglajamR1 ^SSP^	518	99.81–100%	98.46% (*S.* sp. Rod3)
*S. glareoli*	*28S* rRNA	PZ429794	SgraupaukF/SgraupaukR ^GS^	616	99.84–100%	99.68% (*S. jamaicensis*, *S.* sp. Rod7)
*S. glareoli*	*28S* rRNA	PZ429795–PZ429800	GsSstrF1/ GsSstrR1 ^SSN^	540	99.81–100%	99.63% (*S. microti*, *S. jamaicensis*)
*S. halieti*	*ITS1*	PZ429823–PZ429826	GsShalF/ GsShalR2 ^SSP^	596	98.49–100%	96.48% (*S.* sp. isolate Skua-2016-CH)
*S. halieti*	*28S* rRNA	PZ429801–PZ429803	SgraupaukF/SgraupaukR ^GS^	616	99.84–100%	99.19% (*S. corvusi*, *S.* sp. ex *Corvus corax*)
*S. halieti*	*28S* rRNA	PZ429804	GsSstrF1/ GsSstrR1 ^SSN^	540	99.81–100%	99.63% (*S.* sp. ex *Corvus corax*)
*S. kutkienae*	*ITS1*	PZ429827–PZ429829	GsSkutkF2/ GsSkutkR2 ^SSP^	578	99.48–100%	88.76 (*S. cornixi*)
*S. kutkienae*	*28S* rRNA	PZ429805–PZ429806	Ssprod2F/ Ssprod2R ^GS^	618	100%	98.87–99.03% (*S. cornixi*)
*S. turdusi*	*ITS1*	PZ429830	GsSturF/ GsSturR ^SSP^	511	98.25–99.22%	84.77–85.19%
*Sarcocystis* sp. ex *Corvus corax*	*28S* rRNA	PZ429807–PZ429808	SgraupaukF/SgraupaukR ^GS^	616	100%	99.35% (*S. corvusi*)
*Sarcocystis* sp. LT24Sa1	*28S* rRNA	PZ429809	SgraupaukF/SgraupaukR ^GS^	616	N/A	98.37% (*S.* sp. Rod3, *S.* sp. RodES1Av)
*Sarcocystis* sp. LT24Sa1	*28S* rRNA	PZ429810	Ssprod2F/ Ssprod2R ^GS^	619	N/A	98.22–98.38% (*S. glareoli*), 98.38% (*S. jamaicensis*, *S.* sp. RodES1Av)
*Sarcocystis* sp. LT24Sa1	*28S* rRNA	PZ429811	SfunerF/ SfunerR ^SSN^	405	N/A	97.53–97.67% (*S.* sp. Rod3)
*Sarcocystis* sp. LT24Sa11	*28S* rRNA	PZ429812	SgraupaukF/SgraupaukR ^GS^	616	N/A	97.57–97.73% (*S. glareoli*)
*Sarcocystis* sp. LT24Sa11	*28S* rRNA	PZ429813	Ssprod2F/ Ssprod2R ^GS^	619	N/A	97.74–97.90% (*S. glareoli*)
*Sarcocystis* sp. LT24Sa11	*28S* rRNA	PZ429814	SfunerF/ SfunerR ^SSN^	405	N/A	97.52–98.44% (*S. glareoli*)

*—The length of sequences excluding primer binding sites. SSP—species-specific primers targeting detected species, GS—primers capable of identifying multiple *Sarcocystis* spp., SSN—primers designed for a different *Sarcocystis* species that also amplified the detected species, N/A—not applicable. BLAST results are presented with QC (query coverage) values ≥ 95.

**Table 3:** Characterization of Tawny Owl isolates based on light microscopy and molecular analysis.

Owl ID	Microscopic Findings	Species Identified by Molecular Analysis
Sa1	Negative	*Sarcocystis* sp. LT24Sa1 (3)
Sa2	1 sporocyst	*S. glareoli* (3)
Sa3	Negative	*S. glareoli* (3)
Sa4	10 sporocysts	*S. glareoli* (3), *S. halieti* (2)
Sa5	5 sporocysts	*S. halieti* (2)
Sa8	Negative	Negative
Sa9	Negative	*S. glareoli* (3)
Sa10	Negative	*S. kutkienae* (2)
Sa11	Negative	*Sarcocystis* sp. LT24Sa11 (3)
Sa12	2 sporocysts	*S. glareoli* (3)
Sa13	1 sporocyst	*S. glareoli* (3), *S. kutkienae* (2)
Sa14	2 sporocysts	*S. kutkienae* (1)
Sa15	1 sporocyst	*S. glareoli* (3)
Sa16	Negative	*Sarcocystis* sp. ex *Corvus corax* (1)
Sa17	Negative	Negative
Sa19	Negative	Negative
Sa20	6 sporocysts	*S. halieti* (1), *S. turdusi* (1), *Sarcocystis* sp. ex *Corvus corax* (1)
Sa23	Negative	*S. funereus* (1)
Sa24	Negative	Negative
Sa25	3 sporocysts	*S. halieti* (3)
Sa26	2 sporocysts	*S. glareoli* (2)
Sa30	Negative	Negative

Numbers in parentheses indicate the number of primer pairs that successfully amplified each *Sarcocystis* species.

## Data Availability

The *28S* rRNA and *ITS1* sequences of *Sarcocystis* species isolated from intestinal scrapings of Tawny Owls collected in Lithuania are available in the NCBI GenBank database under accession numbers PZ429785–PZ429814 and PZ429815–PZ429830, respectively.
